# Direct electrophysiological evidence for the maintenance of retrieval orientations and the role of cognitive control

**DOI:** 10.1016/j.neuroimage.2018.01.062

**Published:** 2018-05-15

**Authors:** Jane E. Herron

**Affiliations:** Cardiff University Brain Research Imaging Centre (CUBRIC), School of Psychology, Cardiff University, Cardiff, CF24 4HQ, Wales, UK

**Keywords:** Retrieval orientation, Episodic memory, Cognitive control, Post-retrieval processing, Event-related potential, Electrophysiology

## Abstract

Retrieval orientations are memory states that bias retrieval towards specific memory contents. Many neuroimaging studies have examined the influence of retrieval orientations on stimulus processing, but very little direct evidence exists regarding the ongoing maintenance of orientations themselves. Participants completed two memory tasks with different retrieval goals. ERPs were time-locked to a pre-stimulus fixation asterisk and contrasted according to retrieval goals. Pre-stimulus ERPs elicited during the two retrieval tasks diverged at frontal electrode sites. These differences onset early and were sustained throughout the fixation-stimulus interval. The functional and spatiotemporal characteristics of this ERP effect comprise the first direct electrophysiological evidence of the ongoing maintenance of retrieval orientations throughout a task. Moreover, this effect was eliminated in participants who performed a stroop task prior to the memory tests, indicating that reserves of cognitive control play an important role in the maintenance of retrieval orientations throughout memory tasks.

## Highlights

• Pre-stimulus fixation-locked ERPs differed during recognition according to retrieval goal.
• It is proposed that this preparatory effect is a correlate of retrieval orientation maintenance.
• The preparatory effect was eliminated in participants who first completed a stroop task.
• Cognitive control reserves therefore appear to influence retrieval orientation maintenance.
• Elimination of the preparatory effect was associated with enhanced post-retrieval monitoring.

## Graphical abstract

**Figure:**
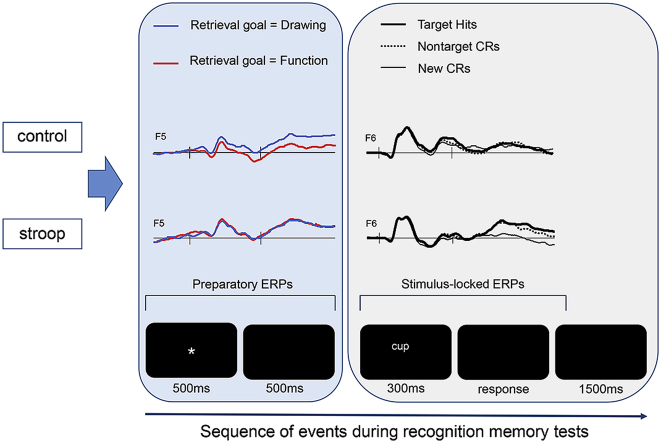

## Introduction

Our episodic memories create the record of our lives, forming a vast library of past experiences rich in sensory, social, emotional and cognitive detail. Researchers are increasingly interested in the ways in which we edit and navigate our memories, searching for desired memories while inhibiting the retrieval of unwanted or irrelevant information. There is now considerable evidence from event-related potential (ERP) and functional MRI studies that cognitive processing during intentional memory retrieval can be oriented towards specific task-relevant features of prior episodes via the adoption of task-specific memory states called ‘retrieval orientations’ (Johnson et al., 1997, Ranganath and Paller, 1999, Rugg et al., 2000, Robb and Rugg, 2002, Herron and Rugg, 2003, Dzulkifli et al., 2004, Herron and Wilding, 2004, Hornberger et al., 2004, Hornberger et al., 2006a, Werkle-Bergner et al., 2005, Hornberger et al., 2006b, Stenberg et al., 2006, Woodruff et al., 2006, Benoit et al., 2009, Bridger et al., 2009, McDuff et al., 2009, Bridger and Mecklinger, 2012, Halsband et al., 2012, Morcom and Rugg, 2012, Rosburg et al., 2013, Rosburg et al., 2014, Roberts et al., 2014, Johnson and McGhee, 2015, Gao et al., 2016, Herron et al., 2016). It is believed that these memory states are maintained for the duration of the requirement to retrieve specific types of contextual information from a prior episode, and that they influence the ways in which incoming stimuli are processed (Rugg and Wilding, 2000).

Neural correlates of retrieval orientations are typically obtained by intermixing previously studied and new items in recognition memory tests, and varying the contextual retrieval requirements throughout these test/s. Retrieval orientations are thought to exert a top-down influence on retrieval stimulus processing to facilitate the retrieval of goal-relevant contextual information (Rugg and Wilding, 2000). This hypothesis predicts that identical retrieval stimuli will be processed differently according to the type of contextual details that participants are attempting to retrieve from the encoding episode. Many studies of retrieval orientation have therefore contrasted neural activity elicited by new items associated with different retrieval demands, as these items should be sensitive to the top-down influence of different retrieval orientations without confounding these with differences in retrieved content. The majority of these studies have obtained neural correlates of retrieval orientation by employing paradigms in which different retrieval demands were imposed in different testing blocks, with the precise spatiotemporal characteristics of the correlates of orientation varying across studies depending on the tasks employed as would be expected of a context-specific effect. Until recently, it appeared that while orientation-related neural differences in retrieval stimulus processing were evident in blocked designs, they were not evident when different retrieval demands were intermixed within the same memory test (Wilding and Nobre, 1999, Werkle-Bergner et al., 2005, Herron and Wilding, 2006, Johnson and Rugg, 2006, Benoit et al., 2009). However, we recently demonstrated that it is possible for participants to flexibly adjust retrieval cue processing in accordance with rapidly changing retrieval orientations if a combination of directed preparatory cues and highly differentiated retrieval tasks are employed (Herron et al., 2016). The cognitive operations reflected by stimulus-locked correlates of retrieval orientation appear to play an important role in memory retrieval, as it has been demonstrated that the magnitude of these correlates are positively correlated with retrieval accuracy in individual differences analyses (Bridger et al., 2009, Rosburg et al., 2011, Rosburg et al., 2014, Bridger and Mecklinger, 2012, Sprondel et al., 2013).

Taking a somewhat different approach, a series of ERP studies from our laboratory capitalised upon the high temporal resolution of the technique by presenting pre-stimulus preparatory cues that directed participants to prepare to retrieve different kinds of contextual information (encoding task or spatial location) upon presentation of the retrieval stimulus (Herron and Wilding, 2004, Herron and Wilding, 2006, Herron et al., 2016). Each preparatory cue type was presented for at least two consecutive trials before switching to a different cue type. Sustained preparatory differences were observed during the cue-stimulus interval according to the retrieval requirements indicated by the cue in all three studies, but the nature of these effects varied with experimental parameters. When participants were required to switch between two source memory tasks, preparatory indices of retrieval orientation were apparent only on the first trial of a particular cue-type (‘switch’ trials) between 700 and 1900ms post-cue at left anterior sites, being absent on the subsequent ‘stay’ trial, and also absent when the two tasks were predominantly blocked (Herron and Wilding, 2006). This functional property suggests that this preparatory correlate of retrieval orientation is related to processes involved in the initial adoption of a retrieval orientation (such as task set configuration), but which are not important for the maintenance of the retrieval state once established (Herron and Wilding, 2006). This preparatory correlate of retrieval orientation was also evident in our earlier study (Herron and Wilding, 2004), but did not onset until the stay trial. As this study also included a third non-episodic task, we proposed that this additional cognitive load may have delayed adoption of the appropriate orientation.

This sustained ERP modulation was replaced by an earlier effect of retrieval cue-type on both switch and stay trials in our most recent study (Herron et al., 2016). This experiment used single non-counterbalanced word questions (e.g. ‘left?’ ‘animacy?’) as preparatory cues that required simple yes/no responses according to whether the stimulus was associated with the source specified by the cue. These retrieval requirements derive from the exclusion task (Jacoby, 1991) in which a ‘target’ source is designated by the experimenter and participants make positive recognition judgments only to items from that source. This is in contrast to the two earlier studies which had used more abstract letters or symbols as cues, and which also required more complex three-way source judgments (e.g. left/right/new). It may therefore be the case that these more constrained and targeted cues allowed retrieval orientations to be initiated more rapidly (Herron et al., 2016). However, it is also possible that these early effects simply reflect perceptual differences between cue-types, an interpretation supported by the fact that this effect was also evident when letters were consistently assigned to cue-types (Herron and Wilding, 2004) but not when counterbalanced symbols were used as cues (Herron and Wilding, 2006). A third potentially explanatory factor is that the two studies in which this earlier effect was observed also included a third task; a semantic task in Herron and Wilding (2004), and a recognition task in Herron et al. (2016). It therefore remains to be seen whether early effects of cue-type are still observed under more constrained retrieval requirements when both visual differences between cue-types and the requirement to switch in/out of a third non-source memory task are removed from the design.

Despite the conceptualisation of retrieval orientations as sustained memory states, direct electrophysiological correlates of their maintenance throughout tasks have proven elusive to date. As described above, ERP correlates of retrieval orientation obtained thus far have been related to i) the initial adoption of an orientation, and ii) the downstream task-dependent processing of stimuli, but direct correlates of the maintenance of the orientation itself have been technically challenging to obtain. Similarly, fMRI studies have reported retrieval orientation effects contingent upon the processing of new items (Hornberger et al., 2006a, Morcom and Rugg, 2012) as well as stimulus-locked effects of retrieval task that are insensitive to retrieval success (Dobbins et al., 2003). Woodruff et al. (2006) reported fMRI data supporting the existence of state-related retrieval orientations by employing a mixed design in which stimulus-related effects were modelled and separated from sustained neural activity that varied in accordance with retrieval goals. The high temporal resolution of ERPs allows for a pre-stimulus time window in which more direct measures of brain activity linked to sustained retrieval orientations can potentially be observed without contamination by stimulus-related effects. Analysis of ERPs recorded during this pre-stimulus window has thus far been restricted to paradigms in which participants switch between different retrieval tasks, but utilising this window in conjunction with blocked retrieval requirements may provide insights into neural activity linked to the maintenance of retrieval orientations.

Retrieval orientations have been linked to the cognitive control of episodic retrieval via the presence of stimulus-locked orientation effects in conjunction with neural evidence of ‘strategic retrieval’ (Herron and Rugg, 2003, Dzulkifli and Wilding, 2005, Dzulkiflil et al., 2006, Morcom and Rugg, 2012, Rosburg et al., 2013, Mao et al., 2017). Strategic retrieval refers to the controlled recollection of task-relevant contextual details alongside a reduction in the recollection of less-relevant memories. Many studies of strategic retrieval borrow from the aforementioned ‘exclusion’ paradigm (Jacoby, 1991), in which items are encoded in at least two different encoding contexts (e.g. two different encoding tasks) and then intermixed with new items in an exclusion memory test. Participants are required to endorse items from a designated encoding context on one response key (‘targets’) and to reject items from the alternate encoding context (‘nontargets’) on the same response key as new items. Neural evidence for strategic retrieval takes the form of significantly larger neural correlates of recollection for targets than for nontargets, these being the ‘left parietal old/new effect’ in ERP studies (i.e. a positive-going shift at left parietal electrode sites for recollected items; (Herron and Rugg, 2003, Dzulkifli and Wilding, 2005, Rosburg et al., 2013, Mao et al., 2017)) and left angular gyrus activation in the fMRI parallel (Morcom and Rugg, 2012). The fact that all of these studies reported neural correlates of strategic retrieval in conjunction with stimulus-locked neural correlates of retrieval orientation indicates that strategic retrieval may be enabled by the maintenance of target-centric retrieval orientations which facilitate the recollection of target memories at the expense of nontarget memories.

Two ERP studies examined the role of cognitive control and working memory capacity (WMC) during strategic retrieval (Elward and Wilding, 2010, Elward et al., 2013). The first study (2010) showed that individual measures of WMC (measured using O-span performance) were positively correlated with ERP indices of recollection and strategic retrieval; the magnitude of the target left parietal effect increased with WMC, and the degree to which the target left parietal effect was larger than the nontarget left parietal effect was also positively correlated with WMC. In a second study (2013), individuals with high WMC who completed a stroop task prior to the memory test exhibited no ERP evidence of strategic retrieval, whereas these were apparent following a control task. The stroop task (Stroop, 1935) requires participants to name the color of the ink in which color names are printed. The two are predominantly incongruous, which means that cognitive control is required to overcome this interference. Because cognitive control is a finite resource, research has shown that taxing these reserves can impair performance on subsequent tasks requiring cognitive control (also referred to as executive function or self-regulation; Baumeister et al., 1998, Muraven and Baumeister, 2000). It has also been shown that autobiographical memory retrieval can be impaired if a stroop task is completed prior to testing (Neshat-Doost et al., 2008, Dahm et al., 2011). Elward et al.’s (2013) additional finding that the ERPs elicited by individuals with high WMC following stroop performance resembled ERPs elicited by individuals with low WMC supported their earlier assertion that WMC plays a role in cognitive control during strategic retrieval (2010). As neither study addressed the impact of stroop performance on neural correlates of retrieval orientation, it is not yet known whether the ability to adopt and/or maintain orientations also depends on the availability of cognitive resources. Given the close theoretical and data-driven links between strategic retrieval and retrieval orientations, this is an important question to resolve.

The principal aims of the present study were therefore twofold. The first was to obtain direct electrophysiological correlates of retrieval orientation maintenance independent of the downstream processing of retrieval stimuli. In order to do this, participants completed two blocked exclusion memory tests with different retrieval demands. Neural activity was time-locked to a neutral pre-stimulus fixation asterisk, recorded for the fixation-stimulus interval, and separated according to retrieval task. By averaging preparatory ERPs across trials throughout each block, any resulting differences due to retrieval task could be attributed to the sustained maintenance of different retrieval orientations elicited by the two tasks independent of retrieval stimulus processing. The second aim was to replicate the stroop manipulation employed by Elward and Wilding, 2010, Elward et al., 2013 to examine the role of cognitive control in participants' ability to maintain retrieval orientations. If retrieval orientations are dependent on cognitive control, as hypothesised, then neural correlates observed in the control group should be attenuated following completion of the stroop task. In addition to examining preparatory ERPs, electrophysiological data time-locked to retrieval stimuli were also analysed. These planned analyses assessed the impact of the stroop manipulation on established ERP correlates of recollection, post-retrieval processing, and stimulus-locked correlates of orientation during strategic retrieval.

## Method

### Participants

Participants were drawn from the undergraduate population studying psychology at Cardiff University. All participants were right-handed native English speakers. They participated on a voluntary basis after giving informed consent in accordance with ethical approval granted by Cardiff University's School of Psychology ethics committee, and were remunerated at a rate of £7.50/hr. Behavioral and electrophysiological data from 50 participants were recorded. Data from two participants were excluded because they failed to contribute at least 16 artifact-free ERP trials to each of the conditions of interest. The remaining 48 participants were allocated to either the stroop or the control group on an alternating basis to minimise order effects. The control group consisted of 24 participants (21 female) aged 18–24 (mean age: 20.4 years) and the stroop group consisted of 24 participants (21 female) aged 18–23 (mean age: 19.9 years; t(1,23) = 1.66, p = 0.11, n.s.).

### Design

**Fig. 1:**
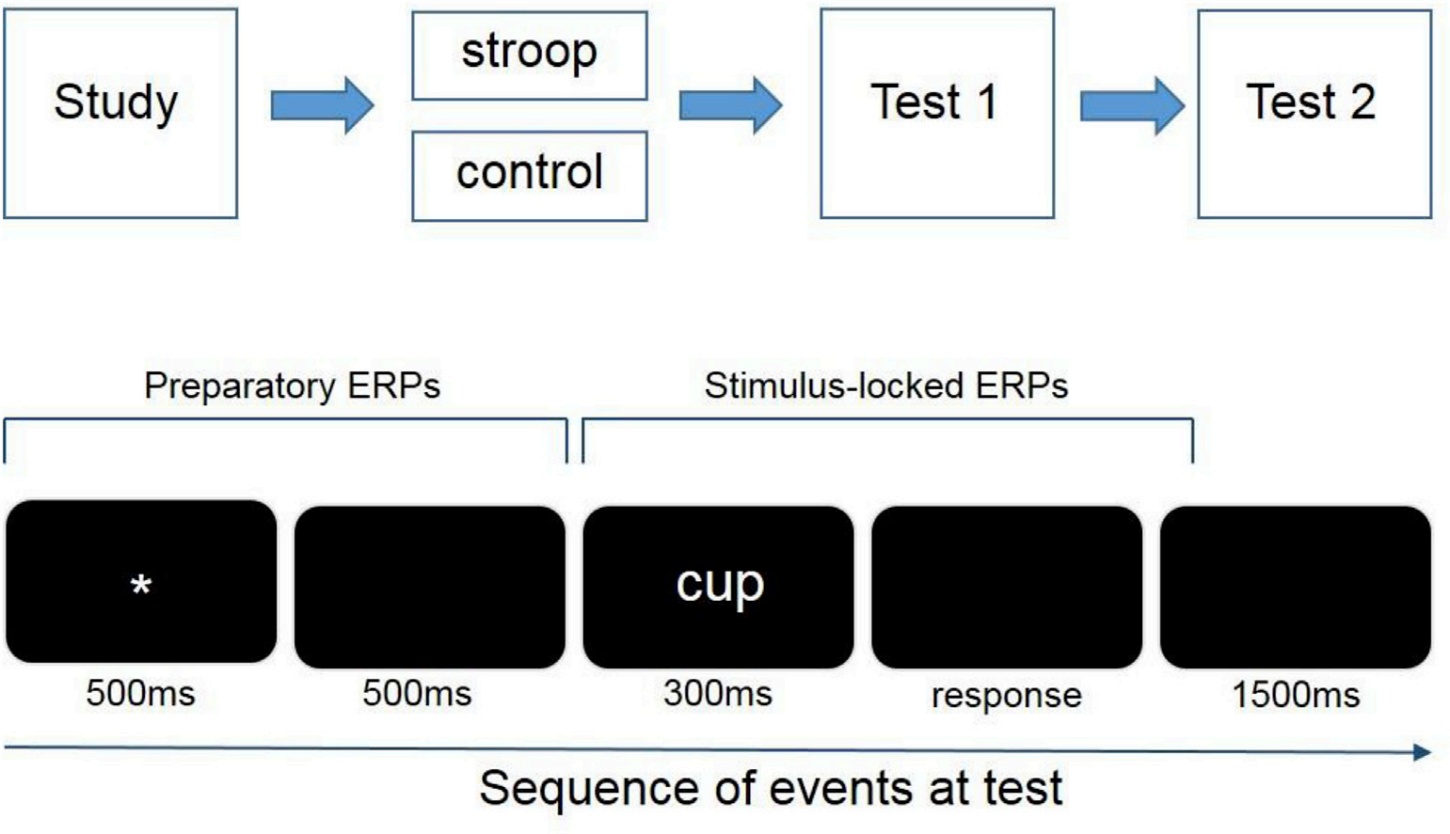
The experimental paradigm is depicted in the upper row and the sequence of events in each test trial is depicted in the lower row.

Stimuli consisted of 240 concrete nouns with a frequency of 1–15 per million and a letter range of 4–9 (Kucera and Francis, 1967). These were presented in white letters on a black background, on a computer screen located 1.2 m from the seated participant. All stimuli were presented at central fixation and subtended maximum visual angles of 0.5° (vertical) and 2.2° (horizontal). The experimental design consisted of a single study phase followed by a 6.5 min stroop/control intervention and concluded with two consecutive exclusion memory tests (see Fig. 1). The pool of 240 words were divided into six lists of 40 words each. The study phase comprised four lists (160 words). Cues preceding each word signalled which encoding task to complete, “FUNCTION?” or “DRAW?”. Cue order was pseudo-randomised with the constraint that no more than three consecutive words were preceded by the same cue. The function task required a verbal response declaring the function of the item (e.g. the item ‘HOUSE’ may elicit a response of ‘to live in’). The drawing task required a verbal response stating whether the item would be easy or difficult to draw. The mapping of stimuli to encoding task was counterbalanced across participants. Cues remained on the screen for 1000 ms, followed by a blank screen for 500 ms. Each study word was then presented for 300 ms before the screen was blanked. Following a verbal response, participants initiated the next trial by pressing a key on a response pad. The next study trial started 2000 ms after this keypress.

The stroop/control intervention followed the method described by Elward et al. (2013). Stroop participants read from 5 A4 pages each of which contained 160 color names (equal numbers of red, green, blue and yellow) arranged in 5 columns for a duration of 6.5 min. Word color and color meaning were incongruent for 75% of items in the stroop group. Participants were asked to name the color of the ink for each word, ignoring word meaning, and reading across the columns. They were instructed to read as many words as possible, but to prioritise accuracy over speed. The protocol was identical for the control group, with the exception that the color names were printed in black ink and participants were instructed to read the words. The two exclusion tasks were then presented consecutively. Each test contained three lists (120 words); one list had been encoded in the function task, the second had been encoded in the drawing task, and the third were new items. Participants were instructed to respond on one key to studied items from a specified encoding task (‘targets’) and to respond on another key both to new items and to studied items from the alternate encoding task (‘nontargets’) with the hand-to-response mapping counterbalanced across participants. One exclusion task specified targets as items encoded in the function task, whereas the other specified targets as items encoded in the drawing task. The presentation order of the two exclusion tasks was counterbalanced across participants. The six wordlists used for the two exclusion tasks were rotated across participants so that each item served as a target, a nontarget and a new item in each of the two exclusion tasks an equal number of times. The sequence of events was identical in both exclusion tasks: each test trial began with a fixation asterisk (500 ms) followed by a blank screen (500 ms) and then the test word (300 ms). The screen was then blanked until the participant responded, and the next trial began 1500 ms later (see Fig. 1).

### EEG acquisition

EEG was recorded using a Biosemi active electrode system from 32 recording locations based on the International 10–20 system (Jasper, 1958) including midline (Fz, Cz, Pz, Oz) and left/right hemisphere locations (FP1/FP2, F7/F8, F5/F6, F3/F4, F1/F2, T7/T8, C5/C6, C3/C4, C1/C2, P7/P8, P5/P6, P3/P4, P1/P2, O1/O2). Additional electrodes were placed on the mastoid processes. Electrooculogram (EOG) was recorded from above and below the left eye (VEOG) and from the outer canthi (HEOG). Electroencephalogram (EEG; range DC-419 Hz; sampling rate 2048 Hz) was acquired referenced to linked electrodes located midway between POz and PO3/PO4, respectively, and was re-referenced off-line to linked mastoids. Data were high-pass filtered off-line (0.03 Hz). Fixation-locked data were down-sampled to 200 Hz, resulting in a total epoch length of 1280 ms including a 255 ms baseline relative to which all mean amplitudes were computed. This epoch spanned the fixation-stimulus interval. Stimulus-locked data were down-sampled to 167 Hz resulting in a total epoch length of 1536 ms including a 102 ms baseline relative to which all mean amplitudes were computed. EOG blink artifacts were identified and corrected using a linear regression estimate (Semlitsch et al., 1986). Trials containing other EOG artifact were rejected, as were trials containing A/D saturation or baseline drift exceeding ±80 mV. A 7-point binomially weighted smoothing filter was applied prior to analysis.

## Results

All behavioral and electrophysiological data were subjected to mixed model ANOVAs which included the Greenhouse-Geisser correction for non-sphericity where necessary (Greenhouse and Geisser, 1959), and epsilon-corrected degrees of freedom are given in the text.

### Behavioral analyses

Accuracy and reaction time (RT) data are shown in Table 1. A mixed model ANOVA conducted on accuracy data incorporated the within-subjects factors of Retrieval Task (function/draw) and Item Type (target/nontarget/new) and the between-subjects factor of Group (control/stroop).

**Table 1:** Mean response accuracy (%) and associated reaction times (ms) to each item type. 95% confidence intervals in parentheses.

	Control		Stroop	
	Accuracy	RT	Accuracy	RT
**Function**				
Target	83 (± 5.0)	802 (± 78)	84 (± 3.4)	734 (± 76)
Nontarget	86 (± 3.3)	861 (± 88)	87 (± 3.5)	846 (± 75)
New	98 (± 1.4)	674 (± 81)	98 (± 1.4)	623 (± 59)
**Drawing**				
Target	78 (± 5.5)	880 (± 98)	81 (± 4.5)	824 (± 79)
Nontarget	84 (± 4.4)	940 (± 110)	85 (± 3.6)	907 (± 110)
New	95 (± 2.9)	707 (± 71)	96 (± 1.8)	682 (± 76)

A main effect of Retrieval Task [F(1,46) = 12.99, p < 0.001, η^2^_p_ = 0.22] reflected higher accuracy in the function task (95% CI = 3.14 ± 1.7). A main effect of Item Type [F(1.7,78.5) = 72.79, p < 0.001, η^2^_p_ = 0.61] was followed up with pairwise comparisons. Main effects of Item Type indicated that accuracy was higher for new items than for either targets [F(1,46) = 99.75, p < 0.001, η^2^_p_ = 0.69, 95% CI = 15.85 ± 3.9] or nontargets [F(1,46) = 112.88, p < 0.001, η^2^_p_ = 0.71, 95% CI = 10.73 ± 2.8], and that accuracy to nontargets was higher than to targets [F(1,46) = 9.83, p = 0.003, η^2^_p_ = 0.18, 95% CI = 5.13 ± 3.8]. No main effects or interactions involving Group were observed (all F's < 1).

A mixed model ANOVA conducted on reaction time data included the within-subjects factors of Retrieval Task (function/draw) and Item Type (correct responses to target/nontarget/new), and the between-subjects factor of Group (control/stroop). A main effect of Retrieval Task [F(1,46) = 10.06, p = 0.003, η^2^_p_ = 0.18] reflected longer RTs when targets were items encoded in the drawing task (95% CI = 67 ± 41). A main effect of Item Type [F(1.8,82.1) = 106.57, p < 0.001, η^2^_p_ = 0.70] was followed up with pairwise comparisons. Main effects of Item Type confirmed that correct responses to targets [F(1,46) = 124.61, p < 0.001, η^2^_p_ = 0.73, 95% CI = 138 ± 24] and nontargets [F(1,46) = 164.74, p < 0.001, 95% CI = 217 ± 33] were slower than those to new items, and that correct responses to nontargets were slower than those to targets [F(1,46) = 26.10, p < 0.001, 95% CI = 79 ± 31]. No main effects or interactions involving Group were observed (all F's < 1).

### ERP analyses

**Fig. 2:**
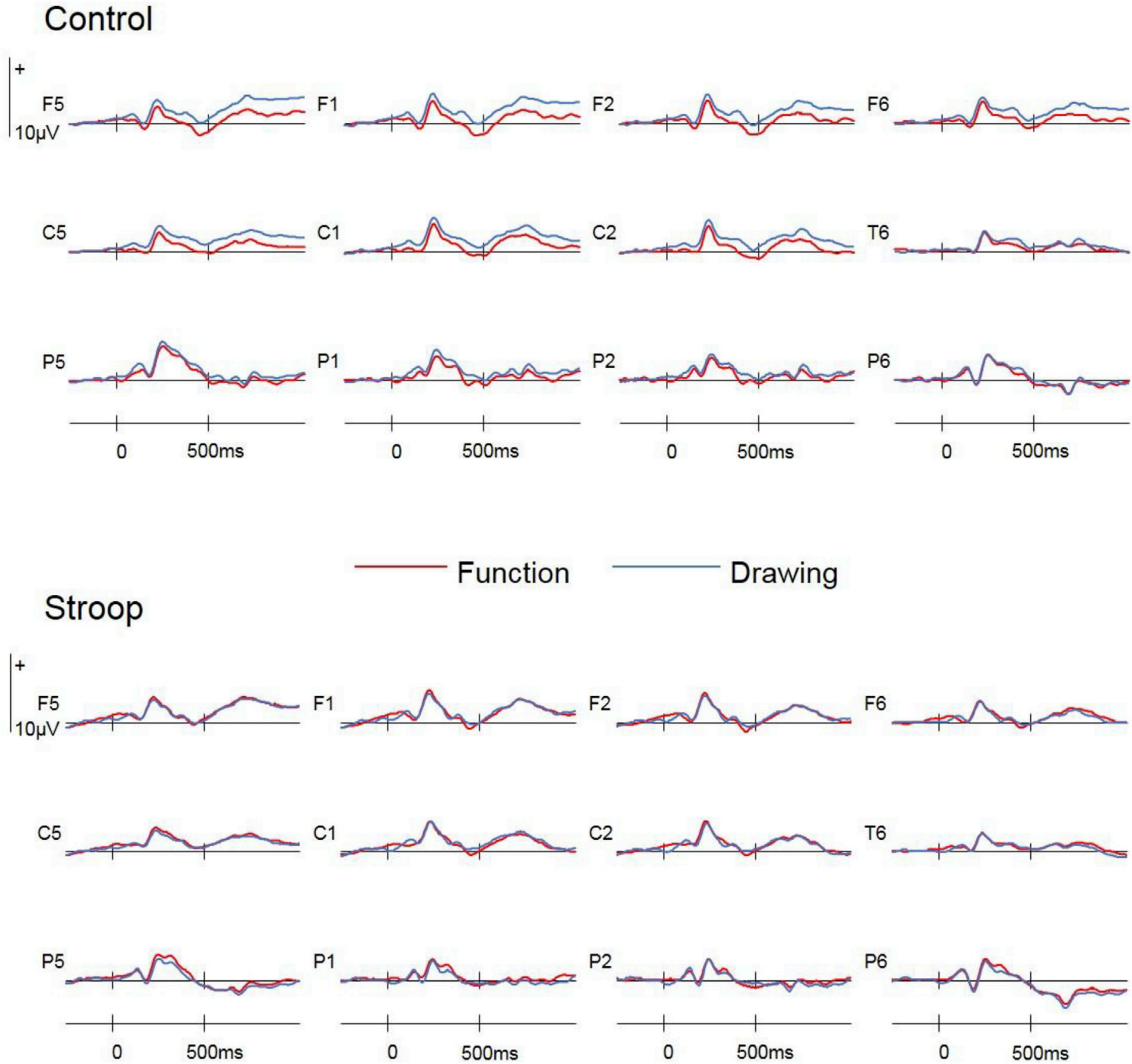
Fixation-locked preparatory ERPs in the Function and Drawing tasks at bilateral frontal (F5, F1, F2, F6), central (C5, C1, C2, C6), and posterior (P5, P1, P2, P6) electrode sites.

Preparatory ERPs time-locked to the fixation asterisk were separated according to Retrieval Task (function/drawing) and Group (stroop/control). These were formed by creating a weighted average of ERPs preceding correct responses to targets, nontargets and new items in each task. The weighted averages were created by weighting averaged ERPs formed for the original conditions by the number of trials contributing to these averages for each participant. Fig. 2 shows that preparatory ERPs in the Drawing task were more positive going at frontal sites than Function ERPs in the control group only, and that these differences onset early and were sustained throughout the fixation-stimulus interval.

The mean numbers of trials (minimum and maximum in parentheses) contributing ERPs for each response type were as follows: control function: 96 (70–114), control drawing: 92 (71–109), stroop function: 98 (72–113), stroop drawing: 94 (67–115). In the absence of *a priori* reasons to select specific sites or latencies, a set of ERP analyses incorporated mean amplitude data from a grid of 24 electrode sites distributed across the scalp (F7/F8, F5/F6, F3/F4, F1/F2, T7/T8, C5/C6, C3/C4, C1/C2, P7/P8, P5/P6, P3/P4, P1/P2) were conducted on data from four successive epochs spanning the fixation-stimulus interval (0–250ms, 250–500ms, 500–750ms, and 750–1000ms), and included the within-subjects factors of Retrieval Task (function/drawing), Anterior/Central/Posterior, Hemisphere and Site (inferior/mid-lateral/superior/midline) as well as the between-subjects factor of Group (stroop/control). Due to the exploratory nature of these analyses, the Benjamini-Hochberg procedure (Benjamini and Hochberg, 1995) for multiple comparisons was applied to analyses conducted for each of the four epochs, and the Benjamini-Hochberg adjusted p-values (against a false discovery rate of 0.05) are reported.

**Fig. 3:**
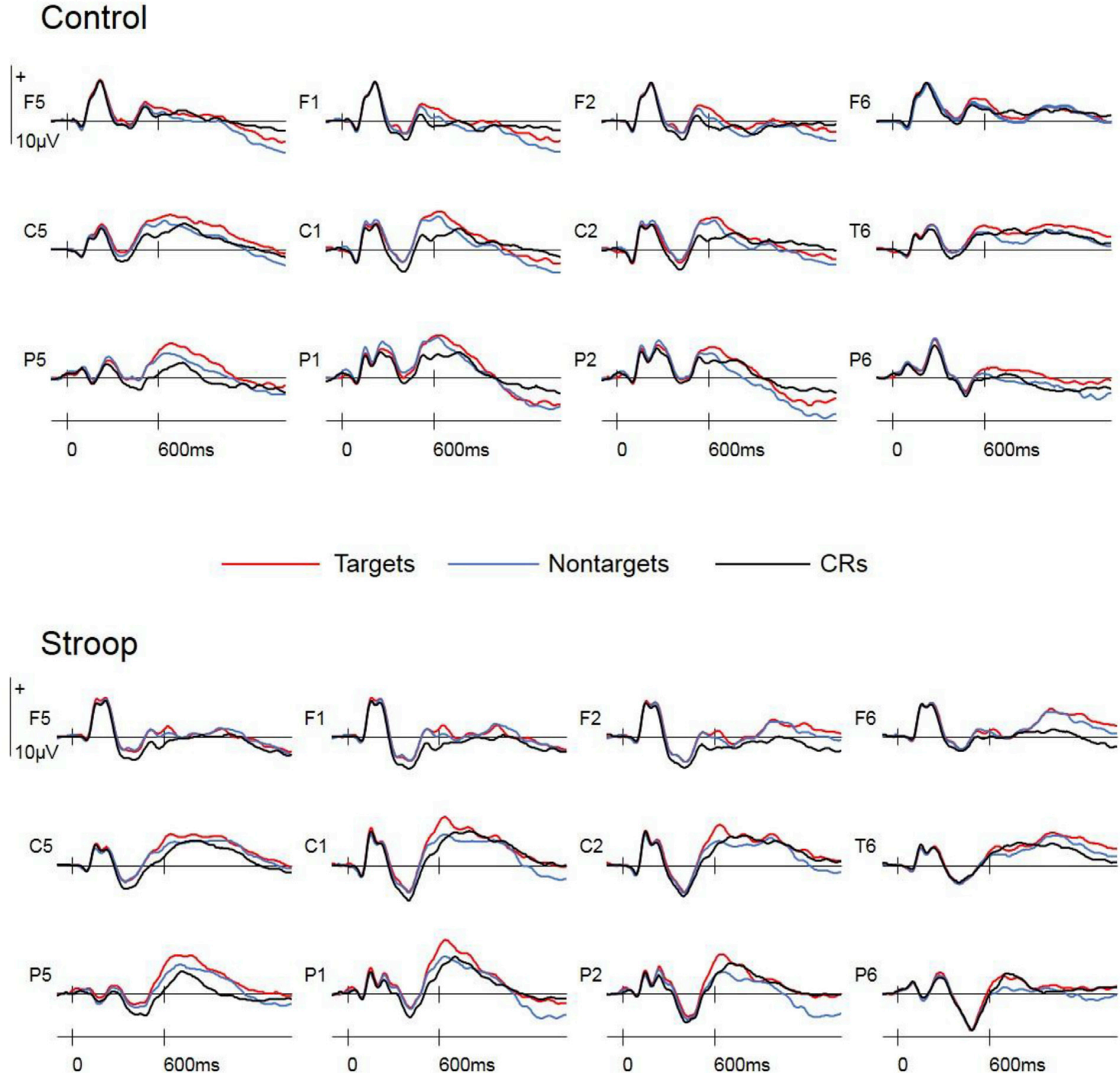
Stimulus-locked ERPs in the control and stroop groups at bilateral frontal (F5, F1, F2, F6), central (C5, C1, C2, C6), and posterior (P5, P1, P2, P6) electrode sites (weighted average of data from function and drawing tasks).

ERPs time-locked to retrieval stimuli associated with correct responses were separated according to Item Type (targets/nontargets/CRs) and Group (stroop/control). Each ERP was a weighted average of data from the Function and Drawing tasks.^1^ Fig. 3 shows that divergences between targets, nontargets and CRs were similar in both groups, with the exception of a late right frontal positivity that was observed for studied items at right frontal sites in the stroop group only.

The mean numbers of trials (minimum and maximum in parentheses) contributing ERPs for each response type were as follows: control targets 62 (38–74), control nontargets 65 (54–76), control CRs 75 (64–79), stroop targets 63 (47–74), stroop nontargets 66 (51–75), stroop CRs 74 (48–79). Planned analyses focused on two ERP old/new effects that have been the subject of extensive investigation (for reviews, see Friedman and Johnson, 2000, Mecklinger, 2000, Wilding and Ranganath, 2011) and which are associated with recollection (the “left parietal old/new effect”) and post-retrieval processing (the “right frontal old/new effect”). These analyses incorporated mean amplitude data from four left parietal electrode sites (P1, P3, P5, P7) between 500 and 800ms and four right frontal electrode sites (F2, F4, F6, F8) between 1110 and 1400ms respectively. Each analysis included the within-subjects factors of Item Type (targets/nontargets/CRs) and Site (inferior/mid-lateral/superior/midline) and the between-subjects factor of Group (stroop/control). Analyses revealing significant effects of Item Type were followed up with three sets of pairwise comparisons; targets/CRs, nontargets/CRs and targets/nontargets.

**Fig. 4:**
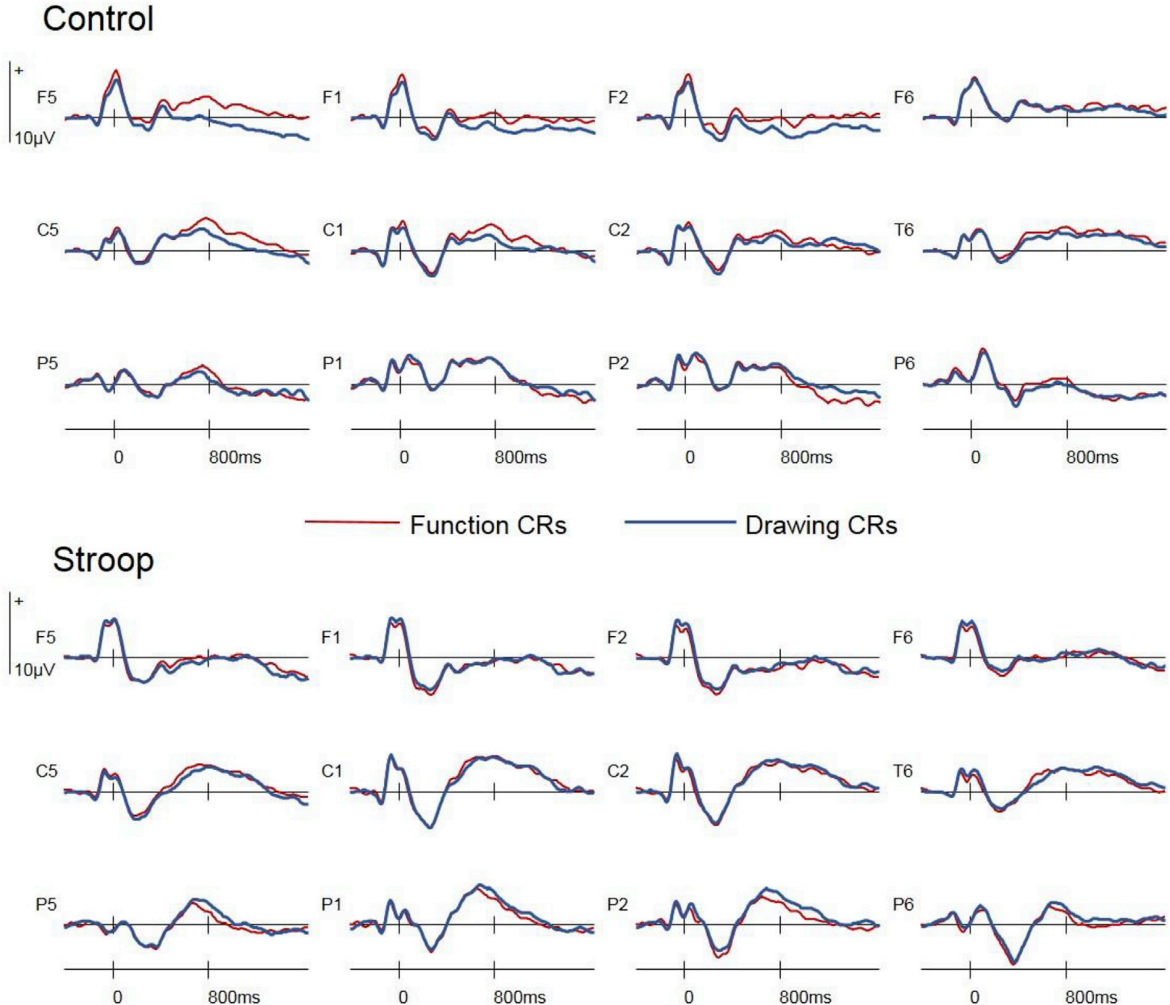
Stimulus-locked CR ERPs in the Function and Drawing tasks at bilateral frontal (F5, F1, F2, F6), central (C5, C1, C2, C6), and posterior (P5, P1, P2, P6) electrode sites.

A further analysis contrasted CRs in the two retrieval tasks to assess stimulus-locked correlates of retrieval orientation and the impact of the stroop intervention on this index. Visual inspection of the ERP data indicated an effect of Retrieval Task at left anterior sites from 500 to 1400ms in the control group only (see Fig. 4), with Function ERPs now eliciting greater positivity than Drawing ERPs. This ANOVA was conducted on data from the same grid of 24 sites described above, measured between 500 and 1400ms as a CR-locked orientation effect for the same pair of retrieval tasks has been reported during this latency region by Bridger et al. (2009). The mean numbers of trials (minimum and maximum in parentheses) contributing ERPs for each response type were as follows: control Function CRs 38 (33–40), control Drawing CRs 37 (27–40), stroop Function CRs 38 (27–40), stroop Drawing CRs 36 (21–40).

#### Preparatory ERPs

Significant interactions between Group x Retrieval Task were observed for each of the four epochs; 0–250ms [F(1,46) = 8.91, BH adjusted p = 0.030, η^2^_p_ = 0.16], 250–500ms [F(1,46) = 7.25, BH adjusted p = 0.030, η^2^_p_ = 0.14], 500–750ms [F(1,46) = 5.06, BH adjusted p = 0.041, η^2^_p_ = 0.10], 750–1000ms [F(1,46) = 6.36, BH adjusted p = 0.030, η^2^_p_ = 0.12]. These interactions reflected larger effects of Retrieval Task in the control group (see Fig. 2). Additionally, a Group × Retrieval Task × Site interaction [F(1.1,52.6) = 3.89, BH adjusted p = 0.049, η^2^_p_ = 0.08] was observed between 0 and 250ms, a main effect of Retrieval Task [F(1,46) = 4.70, BH adjusted p = 0.041, η^2^_p_ = 0.09] was observed between 250 and 500ms, and a Retrieval Task x Anterior/Posterior interaction [F(1.4,64.4) = 5.15, BH adjusted p = 0.030, η^2^_p_ = 0.10] were observed between 750 and 1000ms. The Group x Retrieval Task interactions observed in each epoch were followed up with repeated measures ANOVAs conducted for each group separately. These analyses revealed main effects of Retrieval Task in each epoch for the control group; 0–250ms [F(1,23) = 6.41, BH adjusted p = 0.025], 250–500ms [F(1,23) = 9.90, BH adjusted p = 0.025], 500–750ms [F(1,23) = 6.66, BH adjusted p = 0.025], 750–1000ms [F(1,23) = 6.74, BH adjusted p = 0.025]. Retrieval Task x Anterior/Posterior interactions reflected a frontal maxima between 250 and 500ms [F(1.4,31.6) = 4.41, BH adjusted p = 0.033], 500–750ms [F(1.4,31.3) = 4.77, BH adjusted p = 0.031] and 750–1000ms [F(1.3,30.4) = 6.67, BH adjusted p = 0.025]. A additional Retrieval Task × Site interaction [F(1.2,26.7) = 6.07, BH adjusted p = 0.025] indicated that the effect was also larger towards the midline between 250 and 500ms. No effects of Retrieval Task were observed during any epoch in the stroop group analyses.

**Fig. 5:**
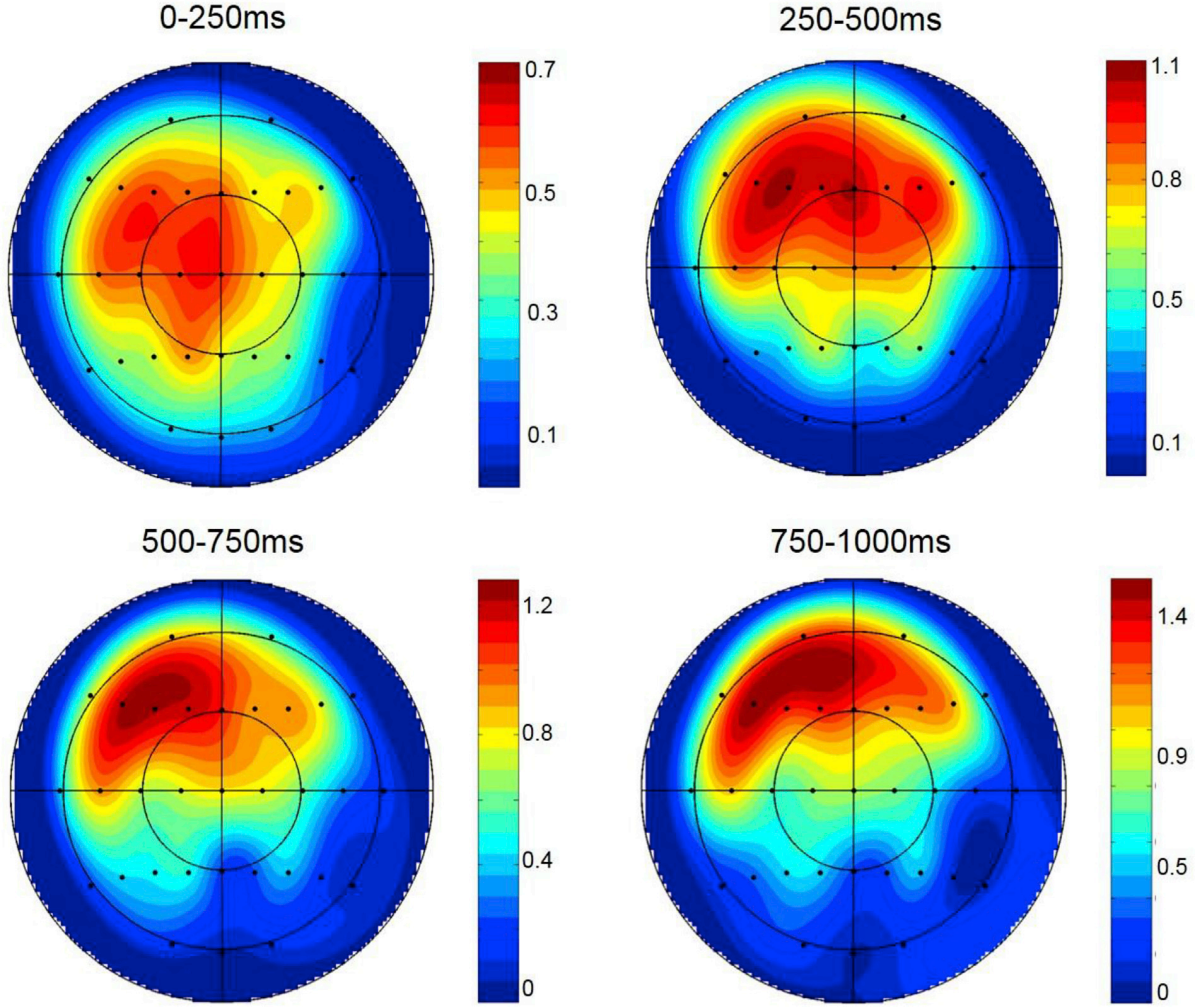
Voltage maps showing the scalp distribution of the ERP effect obtained by subtracting preparatory ERPs in the Function Task from those in the Drawing task at all 32 electrode sites in each of the four epochs.

A topographic analysis was performed on the preparatory effects of Retrieval Task observed for the control group in each epoch to assess whether the same or different neural generators were responsible for this effect across time. This analysis was performed on difference scores formed by subtracting mean amplitudes of preparatory ERPs in the Function task from those in the Drawing task within each epoch, rescaled using the max-min method to avoid confounding changes in amplitude with changes in the shape of scalp distributions (McCarthy and Wood, 1985). The repeated measures ANOVA included the factors of Epoch (0–250ms; 250–500ms; 500–750ms; 750–1000ms), Anterior/Posterior, Hemisphere and Site (inferior/mid-lateral/superior/midline). No effects of Epoch were observed, indicating that the preparatory effects observed within each epoch shared the same scalp distributions (see Fig. 5). Similarly, no effects of Epoch was observed in a second topographic analysis incorporating data from all 32 electrode sites.

Additional analyses contrasted control group trials from the first half of each test block with those from the second half. This was to test the hypothesis that the preparatory correlate of orientation obtained here was related to maintenance rather than adoption, as a large effect at the beginning of each test block (signalling the adoption of orientations) could potentially underlie the effect averaged across the test blocks. If this was the case, then the effect should be absent or attenuated for trials from the second half of each test block. These analyses were identical to those described above for the within-group contrasts, with the additional factor of Trial Order (first/second half) included. These analyses revealed main effects of Retrieval Task in each epoch; 0–250ms [F(1,23) = 6.18, BH adjusted p = 0.021], 250–500ms [F(1,23) = 10.62, BH adjusted p = 0.014], 500–750ms [F(1,23) = 7.39, BH adjusted p = 0.021], 750–1000ms [F(1,23) = 7.45, BH adjusted p = 0.021], with Retrieval Task x Trial Order interactions also evident between 0 and 250ms [F(1,23) = 11.88, BH adjusted p = 0.014] and 250–500ms [F(1,23) = 6.92, BH adjusted p = 0.021]. These interactions reflected significantly larger effects of Retrieval Task in the second half of each test block during both the 0–250ms epoch (first half: 0.02μv, second half: 0.842μv) and the 250–500ms epoch (first half: 0.237μv, second half: 1.149μv). Trial Order did not influence the Retrieval Task effect between 500 and 1000ms. Retrieval Task x Anterior/Posterior interactions between 250 and 500ms [F(1.4,32.8) = 4.94, BH adjusted p = 0.024], 500–750ms [F(1.4,31.7) = 4.43, BH adjusted p = 0.032] and 750–1000ms [F(1.3,30.3) = 6.16, BH adjusted p = 0.021] again reflected the frontal maxima of the effect.

Bivariate correlations (Pearson's R) examined the relationship between the preparatory effect observed in the control group and retrieval accuracy at the level of individual participants. The outcomes of the preceding analyses guided the selection of ERP data from anterior sites (F7, F5, F3, F1, F2, F4, F6, F8) between 0 and 1000ms as an index of the effect. This ERP measure was a difference score obtained by subtracting mean amplitudes in the Function task from those in the Drawing task. This measure was plotted both against target accuracy and target/nontarget discrimination for each participant (collapsed across target designation). No significant correlations were observed between the ERP preparatory effect and either measure of retrieval accuracy.

**Fig. 6:**
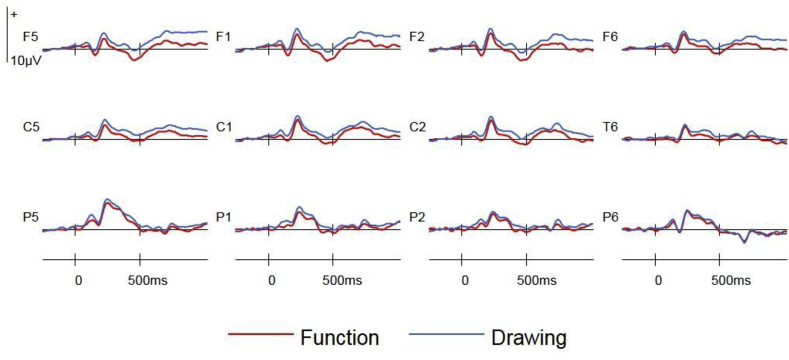
Fixation-locked preparatory ERPs in the accuracy-matched control group (N = 18) at bilateral frontal (F5, F1, F2, F6), central (C5, C1, C2, C6), and posterior (P5, P1, P2, P6) electrode sites.

A final analysis examined the preparatory Retrieval Task effect observed for the control group in a subset of 18 participants for whom target accuracy was statistically equivalent in the Drawing (M = 79, 95% CI = [76.7 to 81.3]) and Function (M = 78, 95% CI = [75.7 to 80.3]) memory tasks. This was achieved by removing the 6 participants who showed the greatest retrieval advantage in the Function (versus Drawing) task, and verifying that target accuracy for the remaining 18 participants did not differ between the two retrieval tasks [t(17) = 0.47, p = 0.647, n.s.]. The preparatory ERP effect for these participants was visually identical to that observed for the whole group (see Fig. 6), and significant main effects of Retrieval Task were obtained for three of the four epochs when subjected to the same analyses; 0–250ms [F(1,17) = 5.34, BH adjusted p = 0.048], 250–500ms [F(1,17) = 7.40, BH adjusted p = 0.038], 500–750ms [F(1,17) = 3.84, BH adjusted p = 0.067, n.s.], 750–1000ms [F(1,17) = 5.05, BH adjusted p = 0.048], as well as a Retrieval Task x Anterior/Posterior interaction [F(1.3,22.8) = 7.06, p = 0.038] between 750 and 1000ms. The preparatory effect was therefore still evident when differences in memory accuracy were eliminated as a potential between-group confound.

#### Stimulus-locked old/new ERPs

The analysis of data from left parietal sites between 500 and 800ms revealed a main effect of Item Type [F(1.9,88.7) = 22.96, p < 0.001, η^2^p = 0.33] and an Item Type × Site interaction [F(3.6,167.8) = 3.00, p = 0.024, η^2^_p_ = 0.06]. A main effect of Item Type [F(1,46) = 38.13, p < 0.001, η^2^_p_ = 0.45] and an Item Type × Site interaction [F(2.1,96.5) = 3.78, p = 0.025, η^2^_p_ = 0.08] was observed in the pairwise comparison of targets and CRs, reflecting greater positivity for targets maximal at P5. A main effect of Item Type [F(1,46) = 17.32, p < 0.001, η^2^_p_ = 0.27] observed in the pairwise comparison between nontargets and CRs reflected greater positivity for nontargets. The pairwise comparison between targets and nontargets revealed a main effect of Item Type [F(1,46) = 8.68, p = 0.005, η^2^_p_ = 0.16] and an Item Type × Site interaction [F(2.4,111.7) = 3.58, p = 0.024, η^2^_p_ = 0.07] reflecting greater positivity for targets maximal at P1. No Group x Item Type interactions were observed in these analyses.

The analysis of data from right frontal sites between 1100 and 1400ms revealed a Group x Item Type interaction [F(1.9,85.2) = 3.29, p = 0.046, η^2^p = 0.07]. A main effect of Item Type [F(1,46) = 4.95, p = 0.031, η^2^_p_ = 0.10] and a Group x Item Type interaction [F(1,46) = 5.27, p = 0.026, η^2^_p_ = 0.10] was observed in the pairwise comparison of targets and CRs, reflecting greater right frontal positivity for targets than CRs in the stroop group only [F(1,23) = 9.72, p = 0.005]. Similarly, Group x Item Type [F(1,46) = 5.77, p = 0.021, η^2^_p_ = 0.11] and Item Type x Site [F(1.8,80.6) = 4.81, p = 0.014, η^2^_p_ = 0.11] interactions observed in the pairwise comparison of nontargets and CRs reflected greater right frontal positivity for nontargets than for CRs in the stroop group only [F(1,23) = 5.93, p = 0.023]. No effect of Item Type were observed in the pairwise comparison of targets and nontargets.

#### Stimulus-locked CR ERPs

The analysis of data from the grid of 24 electrode sites distributed across the scalp between 500 and 1400ms gave rise to an interaction between Retrieval Task x Anterior/Posterior [F(1.4,62.3) = 4.19, p = 0.033], reflecting greater positivity for Function CRs at anterior sites and a smaller effect of reversed polarity at posterior sites (see Fig. 4). No main effects of Retrieval Task were obtained in three post-hoc analyses conducted at anterior, central and posterior sites respectively, and no moderating effects of Group were detected in any analysis.

## Discussion

A large number of ERP and fMRI studies have reported neural correlates of retrieval orientation contingent upon retrieval stimuli processing, and a smaller number of ERP studies have reported preparatory correlates of retrieval orientation linked to their initiation. Direct correlates of the ongoing maintenance of retrieval orientations throughout tasks have, however, proved more elusive. The initial aim here was to obtain novel electrophysiological evidence of the maintenance of retrieval orientations throughout tasks that were not contingent upon the processing of retrieval stimuli. For the first time, preparatory ERPs were formed for the fixation-stimulus interval by averaging across trials throughout blocked retrieval tasks with consistent retrieval goals, and these were found to differ as a function of retrieval task in a control group. Preparatory ERPs in the two tasks diverged early after fixation onset, and resembled previously reported preparatory correlates of retrieval orientation in two key ways; i) the differences were characterised by slow wave activity that was sustained and topographically equivalent across epochs throughout the fixation-stimulus interval, and ii) the effect was maximal at frontal electrode sites. The early and sustained nature of the effect, combined with the consistent retrieval requirements imposed, supports the view that this event-related effect is capturing retrieval orientations that are tonically maintained throughout retrieval tasks.

Previously, preparatory correlates of retrieval orientations have been formed for cues which explicitly direct participants to proactively prepare to retrieve the designated contextual details (Herron and Wilding, 2004, Herron and Wilding, 2006, Herron et al., 2016). As such, indices of orientation obtained in these earlier paradigms are likely to contain an array of processes linked to the adoption of a new orientation, disengagement of the previous orientation, the mapping of cue-type to retrieval requirements, and more general cognitive control processes. In contrast, time-locking to a neutral fixation asterisk under blocked retrieval requirements enabled the capture of neural activity linked to the maintenance of a retrieval orientation throughout a task in the absence of active updating of retrieval requirements and switching between memory tasks. The effect between 500 and 1000ms was not sensitive to whether trials came from the first or second half of the test blocks, which is consistent with a role in maintenance rather than adoption. However, the early portion of the effect (0–500ms) increased in magnitude from the first to the second half of the test blocks. While the direction of this effect of trial order is clearly inconsistent with an adoption account, it does suggest that very early preparatory processes related to orientation are sensitive to the amount of time that the same retrieval goal has been maintained, with task-specific neural activity diverging more rapidly post-fixation the longer that an orientation has been maintained. This in turns suggests that the neural implementation of orientation maintenance may fluctuate in accordance with external environmental stimuli (in this case, a pre-stimulus fixation signalling an upcoming memory probe) and/or internal factors such as attention or time-on-task.

How do these findings relate to indices of stimulus-independent retrieval orientation reported in other studies? When considering this question, it is important to note that neural correlates of orientation are likely to differ across studies according to the specific retrieval requirements employed. Nonetheless, the spatiotemporal characteristics of the effect reported here are strikingly similar to those of the preparatory ERP effect linked to the initiation of retrieval orientations (Herron and Wilding, 2004, Herron and Wilding, 2006). Both effects are characterised by sustained slow wave activity maximal over frontal electrode sites, and the scalp distribution of the effect obtained here visually resembles the left-lateralised effect reported previously (see Fig. 2, Fig. 5) although this lateralisation was not statistically significant. These similarities suggests that regions involved in the initiation of retrieval orientations may overlap with those responsible for their maintenance. Interestingly, Herron & Wilding (2006) reported an experiment in which the two retrieval tasks were predominantly blocked yet no preparatory effects of cue-type were observed (Experiment 2). Participants in this experiment, however, were still encouraged to attend to cues signalling the kind of contextual information to be retrieved by the insertion of ‘catch’ trials of the alternate cue-type, and this may have prevented them from fully adopting appropriate retrieval orientations.

Although the poorer temporal resolution of fMRI does not easily facilitate time windows of stimulus-independent brain activity, it is possible to employ mixed designs which model stimulus-related neural activity and separate this from sustained neural activity likely to reflect memory states (Otten et al., 2002, Visscher et al., 2003). Woodruff et al. (2006) used this approach to identify brain regions linked to the supra-item maintenance of retrieval orientations independent of stimuli processing. Retrieval goals were items studied either as words or as pictures, and activations linked to orientations targeting these different contents were observed in medial and lateral prefrontal cortex amongst other regions. Importantly, this study also dissociated regions associated with the sustained maintenance of retrieval orientations from those involved in the differential processing of new items in the two retrieval tasks, evidencing the view that the use of new item contrasts as a proxy for retrieval orientations are identifying the downstream consequence of retrieval orientations rather than the orientations directly. A second event-related fMRI study used preparatory cues to specify whether encoding list or encoding task was to be retrieved, and found differential activation in left lateral anterior prefrontal cortex which peaked 4s prior to recollection effects and which was apparent even in the absence of retrieval stimuli (Simons et al., 2005). The fact that this design required participants to switch frequently between retrieval tasks suggests that this correlate of retrieval orientation is more likely to reflect adoption than maintenance, and Simons et al. (2005) proposed that activity in this region may have given rise to the preparatory ERP orientation effect originally reported by Herron and Wilding (2004).

The second aim was to test the hypothesis that depleting participants' reserves of cognitive control by asking them to complete a stroop task would reduce their ability to maintain retrieval orientations. This hypothesis was motivated by the finding that individuals with high levels of working memory capacity show a reduction in ERP measures of strategic retrieval following completion of the stroop task (Elward et al., 2013). Because strategic retrieval (as evidenced by smaller left parietal effects for nontargets than for targets) is thought to be supported by target-focused retrieval orientations (Dzulkifli and Wilding, 2005, Dzulkiflil et al., 2006), the logical inference is that correlates of these retrieval orientations may likewise be reduced following stroop completion. The results supported this hypothesis, with the retrieval orientation effect obtained in the control group being neither visually nor statistically evident in the group completing the stroop task. Reserves of cognitive control therefore appear to influence the ability to maintain an appropriate retrieval orientation. Further research in which these measures of retrieval orientation are correlated with individuals' working memory scores would provide insight into the specific role of working memory capacity in the maintenance of retrieval orientations.

The contrast between CRs from the two retrieval tasks also initially appeared to indicate a role for cognitive control in this downstream index of retrieval orientation, with an effect of retrieval goal visually evident for the control group only. However, the interaction between group and this effect did not reach statistical significance. Indeed, the CR-locked effect itself (independent of group) was not robust, statistically evident only as a crossover interaction with the anterior/posterior axis, with the main effect of retrieval task failing to reach significance at any level of this axis. The influence of cognitive control on stimulus-locked correlates of retrieval orientation therefore remains ambiguous and requires further investigation with a task pair optimised for this particular contrast. While this lack of robust statistical evidence precludes detailed discussion of this aspect of the data, the observation that the preparatory and stimulus-locked indices of orientation shared a left anterior scalp distribution raises the possibility that common brain regions may be involved in both stages of strategic retrieval, although the reversal in polarity between the two effects indicates that they are not indexing exactly the same process. Examining the relationship between these two measures will therefore be a future research goal.

Correct responses to nontargets were slower than those to targets in both groups, supporting the view that all participants strategically prioritised the retrieval of targets. This is consistent with a recent meta-analysis of strategic retrieval ERP studies which reported that nontarget RTs are delayed relative to nontargets only when target information is strategically prioritised (Rosburg and Mecklinger, 2017). The absence of an interaction between Retrieval Task and target/nontarget reaction times confirms that this was a general strategic effect rather than differences in retrieval time for items from the Function and Drawing tasks. Converging with these behavioral findings, the analysis of stimulus-locked ERPs indicated that ERP indices of strategic retrieval were evident and equivalent in both groups. Strategic retrieval does not therefore appear to be causally dependent on the maintenance of retrieval orientation. It is perhaps surprising that stroop completion did not reduce ERP measures of strategic retrieval, given Elward and Wilding's (2010) proposal that strategic retrieval only occurs when sufficient resources are available for cognitive control of retrieval. The degree to which stroop completion influences strategic retrieval has been shown to be highly variable, with only those participants with higher levels of WMC (as indexed by the O-span task) showing this reduction (Elward et al., 2013). As both participant groups in the present study likely comprised a mixture of high and low WMC individuals, the influence of the stroop manipulation on measures of strategic recollection may have been too variable to be detected.

Similarly, no significant effects of stroop completion were detected in the behavioral analyses. This is reminiscent of Elward et al.’s finding that response accuracy did not predict ERP measures of strategic retrieval (2010, 2013). These authors proposed that the availability of cognitive resources may not necessarily lead to optimal retrieval processing, and also suggested that the exclusion task may not be sufficiently sensitive to stroop-induced changes in response accuracy, noting that stroop completion did decrease the number of details recalled in an autobiographical memory test (Neshat-Doost et al., 2008) indicating that other measures of retrieval accuracy may be more sensitive to this manipulation. Converging with the group analyses, the correlational analyses also indicated that the magnitude of the preparatory orientation effect did not predict retrieval accuracy at the level of individual participants. In the absence of group effects on either memory performance or ERP indices of strategic retrieval, what benefits did maintaining retrieval orientation bring to the control group in the present study? The most obvious answer comes from the planned analysis of stimulus-locked ERPs from right frontal sites, which revealed that correctly classified targets and nontargets elicited significantly more positive-going ERPs than new items in the stroop group, an effect that was absent in the control group. This old/new effect (the ‘right frontal old/new effect’) is thought to reflect post-retrieval monitoring processes that evaluate retrieved information (Johnson et al., 1997, Donaldson and Rugg, 1998, Cruse and Wilding, 2009, Wilding and Ranganath, 2011), and has specifically been linked to the monitoring of episodic memory as it is larger when source judgments are required (Johnson et al., 1997, Senkfor and Van Petten, 1998, Van Petten et al., 2000, Johansson et al., 2002) and larger for correct than incorrect source judgments (Wilding and Rugg, 1996; but see Hayama et al., 2008 for a more general monitoring account). Within this framework, the stimulus-locked ERPs indicate that participants in the control group were able to achieve equivalent levels of memory accuracy without recourse to the additional post-retrieval monitoring processes evident in the stroop group. It therefore appears that maintaining retrieval orientations throughout a memory task reduces the need for post-retrieval monitoring, and that monitoring processes were engaged by the stroop group to compensate for the failure to maintain target-specific retrieval orientations.

In conclusion, direct ERP evidence for the supra-item maintenance of retrieval orientations was obtained by time-locking neural activity to neutral pre-stimulus fixation asterisks during two different retrieval tasks. This took the form of an early-onsetting and sustained differentiation due to retrieval goals that was maximal at frontal electrode sites and significant throughout the fixation-stimulus interval. This correlate of retrieval orientation was evident only in the control group, and appeared to be entirely eliminated in the group who completed a stroop task prior to retrieval. This finding suggests that available reserves of cognitive control play an important role in the maintenance of retrieval orientations. While no significant between-group differences were observed in memory accuracy, reaction times, or ERP indices of strategic retrieval, between-group differences at right frontal electrode sites in the stimulus-locked ERPs indicated that the ability to maintain retrieval orientations reduced the need for post-retrieval monitoring.

## Funding

This work was supported by the Wellcome Trust [grant number 106278/Z/14/Z].

## Footnotes

1 Retrieval Task did not moderate effects of Item Type when included as a factor in the planned analyses, therefore ERPs were collapsed across this factor.
